# HIV-1, methamphetamine and astrocytes at neuroinflammatory Crossroads

**DOI:** 10.3389/fmicb.2015.01143

**Published:** 2015-10-27

**Authors:** Kathleen Borgmann, Anuja Ghorpade

**Affiliations:** Department of Cell Biology and Immunology, University of North Texas Health Science CenterFort Worth, TX, USA

**Keywords:** neuroinflammation, astroglia, HIV, methamphetamine, neurocognitive impairment

## Abstract

As a popular psychostimulant, methamphetamine (METH) use leads to long-lasting, strong euphoric effects. While METH abuse is common in the general population, between 10 and 15% of human immunodeficiency virus-1 (HIV-1) patients report having abused METH. METH exacerbates the severity and onset of HIV-1-associated neurocognitive disorders (HAND) through direct and indirect mechanisms. Repetitive METH use impedes adherence to antiretroviral drug regimens, increasing the likelihood of HIV-1 disease progression toward AIDS. METH exposure also directly affects both innate and adaptive immunity, altering lymphocyte numbers and activity, cytokine signaling, phagocytic function and infiltration through the blood brain barrier. Further, METH triggers the dopamine reward pathway and leads to impaired neuronal activity and direct toxicity. Concurrently, METH and HIV-1 alter the neuroimmune balance and induce neuroinflammation, which modulates a wide range of brain functions including neuronal signaling and activity, glial activation, viral infection, oxidative stress, and excitotoxicity. Pathologically, reactive gliosis is a hallmark of both HIV-1- and METH-associated neuroinflammation. Significant commonality exists in the neurotoxic mechanisms for both METH and HAND; however, the pathways dysregulated in astroglia during METH exposure are less clear. Thus, this review highlights alterations in astrocyte intracellular signaling pathways, gene expression and function during METH and HIV-1 comorbidity, with special emphasis on HAND-associated neuroinflammation. Importantly, this review carefully evaluates interventions targeting astrocytes in HAND and METH as potential novel therapeutic approaches. This comprehensive overview indicates, without a doubt, that during HIV-1 infection and METH abuse, a complex dialog between all neural cells is orchestrated through astrocyte regulated neuroinflammation.

## Introduction

### Burden of HIV-1 and HAND

Worldwide an estimated 33 million people are infected with human immunodeficiency virus (HIV) and without effective treatment, HIV results in a progressive failure of the immune system. Approximately 1.1 million Americans are currently living with HIV or acquired immune deficiency syndrome (AIDS), with an estimated 50,000 new infections occurring in the U.S each year^1^. While the age at which neurocognitive issues develop is increasing with antiretroviral therapy (ART), ~69% of HIV+ patients continue to develop HIV-1-associated neurocognitive disorders (HAND; Matinella et al., 2015). Although the prevalence of HIV-1-associated dementia (HAD) has decreased from ~20% to less than 5% with wide-spread use of ART, other neuropsychiatric complications of HIV, including delirium, neurobehavioral impairments (depression), asymptomatic neurocognitive impairment (ANI) and minor neurocognitive disorder (MND) remain prevalent (McArthur et al., 2005; Robertson et al., 2007; Matinella et al., 2015). Significant glial activation can be found in brain tissues even in the absence of HIV encephalitis (HIVE) or even active viral replication, implicating inflammation as a causative mechanism of HAND (Tavazzi et al., 2014).

### Burden of METH abuse

Abuse of the potent psychomotor stimulant methamphetamine (METH) remains a significant public health concern as it results in neurotoxic outcomes including deficits in memory, executive function, anxiety, depression, psychosis and other neurologic manifestations (Cadet and Krasnova, 2009; Nagai and Yamada, 2010; Rusyniak, 2013). Despite declining use since 1999, 1.2 million people reported METH use in 2012, 133,000 of which were new users aged 12 and older^2^. An urban men's health study of over 2000 men who have sex with men (MSM) indicates that use of METH and other stimulants by MSM is 10 times greater than the general population and METH abusers are 20% more likely to contract sexually transmitted diseases, including HIV-1 (Stall et al., 2001; Rosenthal, 2006)^3^. METH administration occurs by various routes including oral, snorting, smoking and intravenous injection. The associated euphoria due to neurotransmitter release disappears before drug concentrations in the blood fall significantly, leading to “binging and crash” patterns of abuse, tolerance and increased METH intake^2^. Chronic METH exposure leads to a variety of adverse physiological consequences including skin lesions, tooth decay, weight loss, altered immunity, and cognitive impairment. It has been estimated that 40% of METH users exhibit global neuropsychological impairment (Rippeth et al., 2004).

### METH and HIV-1 comorbidity

Eight percent of newly diagnosed HIV-1 infections are attributed to intravenous drug use and the National Institute on Drug Abuse reports that 25% of diagnosed HIV-1-infected individuals report treatment for the use of drugs and alcohol^4^. While accurate statistics documenting METH abuse in HIV-1-infected individuals are not available, studies show that METH use exacerbates HIV-1 infection, accelerating the severity and onset of HAND, along with immune dysfunction and resistance to ART therapy (reviewed in Passaro et al., 2015) Studies report that 53–58% of HIV+ METH users exhibit neurocognitive impairment compared to 40% in either HIV+ or METH+ alone; however, their interaction is poorly understood (Rippeth et al., 2004; Gupta et al., 2011). In part, the neurological complications in both METH abuse and HAND are associated with increased permeability of the blood brain barrier (BBB) and neuroinflammation. These are mediated through cellular and molecular mechanisms such as gliosis, viral replication, oxidative stress and excitotoxicity (Rippeth et al., 2004; Ramirez et al., 2009; Potula et al., 2010; Sharma et al., 2011; Cisneros and Ghorpade, 2012).

The study of inflammation generally focuses on the contributions of professional immune cells. However, the unique nature of the brain as an immune privileged site implicates neural cells in the regulation of immune responses. Glia, specifically astroglia and microglia, account for 50–80% of the cells in the brain, outnumbering neurons in certain regions by as much as 10:1 (Dobbing and Sands, 1973; Kandel et al., 2000; Azevedo et al., 2009). As the resident immune cells of the brain, microglia are accountable for brain surveillance and immunity, while astrocytes have a significant repertoire of immune functions that complement their “neural” functions. Astrocytes, through regulation of neuroinflammation, are implicated in neurodegenerative diseases such as Alzheimer's disease (AD; Roth et al., 2005; Weinstein et al., 2013), hepatic encephalopathy (Coltart et al., 2013), multiple sclerosis (MS; Brosnan and Raine, 2013; Kostic et al., 2013), epilepsy (Devinsky et al., 2013), amyotrophic lateral sclerosis (Evans et al., 2013), Parkinson's disease (PD; Tufekci et al., 2012), aging and depression (Paradise et al., 2012) and HAND (Borjabad et al., 2010; Cisneros and Ghorpade, 2012). Reactive glia participate in neuroinflammation by synthesizing and releasing various powerful pro-inflammatory and neuroactive substances, like cytokines, chemokines, nitric oxide (NO), glutamate, reactive oxygen species (ROS), neurotrophins and transforming growth factors (TGF; da Cunha and Vitkovic, 1992; Chiueh and Rauhala, 1999; Wang et al., 2004; Hult et al., 2008; Fitting et al., 2012; Ramesh et al., 2013; Salamanca et al., 2014). Although astroglia play a central role in HIV-1-associated neuropathogenesis, serving as reservoirs for latent HIV infection, chronic inflammation and as sources of neurotoxicity during HAND. There is a paucity of information regarding the mechanisms at play during HIV-1 and METH comorbidity. Due to the addictive nature of METH abuse, METH interactions with neurons leading to dopamine release and subsequent toxicity have been a focus of much investigation. However, despite apparent glial activation, the mechanisms through which METH interacts with glia to alter astrocyte and microglial function are much less apparent (Chiu and Schenk, 2012; Cisneros and Ghorpade, 2012; Friend and Keefe, 2013). A better understanding of astrocyte regulation of HIV-1 and METH-mediated neurodegeneration would help identify mechanistic targets coordinating glial activation. By therapeutically reducing acute and chronic inflammation, neurological impairments such as HAND could be ameliorated or even prevented.

## Astrocytes in HAND

As a predominant cell in the brain, astrocytes regulate the central nervous system (CNS) physiological environment at both the BBB and in the parenchyma. As integral members of the BBB, astroglia respond to immunomodulatory signals including, but not limited to, cytokines and prostaglandins. During HIV-1 CNS infection, the BBB integrity is compromised thus permitting the peripheral immune system to trigger neuroinflammation and oxidative stress. Astrocytes secrete a variety of neuroactive molecules in response to HIV-1- and METH-associated stimuli. In this manner, astrocytes regulate the physiological functions of neural cells in their immediate vicinity and cells within the reach of their many foot processes. As neuroinflammation persists, the ability of astrocytes to regulate BBB integrity, and neurotransmission in tripartite synapses is impaired. Under chronic disease, astrocyte expression of critical transporters and enzymes that clear neurotransmitters, neutralize ROS and balance ECM remodeling dwindles to levels where homeostasis is no longer sustainable. Eventually, neuronal function and survival are impaired due to insufficient support and direct toxicity. Taken together, astrocyte dysfunction during METH abuse, in the setting of HIV-1 infection, contributes both to chronic BBB damage and propagation of a CNS environment dominated by inflammation, oxidative stress, and excitotoxic insults, that culminate in neurodegeneration.

### Blood brain barrier

In the absence of trauma, infection or disease, and despite fenestration of the BBB in specific areas, the majority of the brain is sealed to peripheral immune surveillance Astrocyte foot processes cover tight junctions between brain microvascular endothelial cells (BMVECs). Astrocyte foot processes also traverse the basal lamina to physically interact with BMVEC, assist with BMVEC differentiation and provide biochemical support (Hamm et al., 2004; Ivey et al., 2009). In fact, in a coculture study, simply removing astrocytes was sufficient to cause tight junction opening and increased BBB permeability (Hamm et al., 2004). Multiple membrane proteins seal tight junctions, including claudin, occluding, and junctional adhesion molecules, while accessory proteins such as zonula occludens (ZO)-1/2 link these tight junction proteins to the BMVEC actin cytoskeleton. The expression and function of several key tight junction proteins are altered during HIV-1 infection and METH exposure leading to increased BBB permeability and viral neuroinvasion (Atluri et al., 2015; Northrop and Yamamoto, 2015).

The Trojan horse model of HIV-1 infection postulates, that early during infection circulating monocytes cross the BBB and carry virus into the CNS. Recent longitudinal studies indicate that the BBB then reseals or compartmentalizes the CNS HIV-1 infection. In ART naïve subjects HIV-1 replication and evolution proceeded independent from the periphery during the first 2 years of infection (Sturdevant et al., 2015). Further, cerebrospinal fluid (CSF) samples showed that compartmentalized HIV-1 replication correlated with a marked inflammatory response in the CSF indicative of ongoing or recurrent neuroinflammation (Sturdevant et al., 2015). In both the periphery and the CNS, HIV-1-infected cells express viral proteins, including glycoprotein (gp)120, transactivator of transcription (Tat) and negative regulator factor (Nef), along with elevated levels of a host of inflammatory mediators such as cytokines and chemokines. These act alone, or in concert, to damage the integrity of the BBB. METH exposure has been shown to increase BBB permeability to small molecules by regulating both tight junction protein expression and intracellular vesicular transport (Dietrich, 2009; Salamanca et al., 2014). METH is capable of traversing the BBB without assistance and thus can act upon the BBB in the periphery and CNS concurrently. METH activates lymphocytes and promotes transendothelial migration (Martins et al., 2013). METH also increases HIV-1 replication in lymphocytes and increases HIV-1 receptor expression on dendritic cells (Liang et al., 2008; Nair et al., 2009; Cen et al., 2013; Atluri et al., 2015). Further, METH exposure suppresses anti-HIV-1 activity in macrophages (MP) by downregulating toll-like receptor-9 expression. Decreased interferon (IFN)-α expression by METH-exposed MP impairs innate host immunity against HIV-1 (Cen et al., 2013). Together, METH and HIV increase BBB compromise and immune cell infiltration to increase neuroinflammation.

Since interactions between circulating immune cells and BMVECs are mediated through cytokines, chemokines and adhesion molecules; alterations in viral proteins and chemokines play an important role in regulating leukocyte extravasation through the BBB during HIV-1 CNS infection. Cells on either side of the BBB participate in the dialog, including circulating leukocytes, BMVEC, perivascular MP, microglia and astrocytes (Langford and Masliah, 2001; Strazza et al., 2011; Louboutin and Strayer, 2012; Woollard et al., 2014). BMVEC expression of cell adhesion molecules is increased by leukocyte binding or by cytokines, such as interleukin (IL)-17, tumor necrosis factor (TNF)-α, IFN-γ, IL-22, and IL-1β, from activated leukocytes, microglia and astrocytes. Activated leukocytes penetrate the BBB by interacting with cell adhesion molecules on BMVEC (Cayrol et al., 2008; Alvarez et al., 2011; Elahy et al., 2015). HIV-1 proteins Tat and gp120 are directly toxic to BMVECs, affecting expression of tight junction proteins, ZO-1, claudin-5 and occludin, and matrix metalloproteinases (MMP; Langford and Masliah, 2001; Strazza et al., 2011; Louboutin and Strayer, 2012; Woollard et al., 2014). Similarly, METH enhances BBB permeability by modulating tight junction protein expression in BMVECs. METH exposure alone significantly decreases the percentage of BMVEC expressing ZO-1, while increasing the percent expressing JAM-2. Combined treatment with gp120 decreased expression of tight junction proteins compared to control (Mahajan et al., 2008). In an *in vitro* BBB model, METH exposure significantly increased transmigration of peripheral blood mononuclear cells (PBMCs) in response to a CCL5 chemotactic gradient compared to unexposed controls. The transmigration of HIV-1-infected PBMCs increased significantly compared to control PBMCs and doubled upon METH exposure, as compared to HIV-1 alone (Mahajan et al., 2008).

The entry of HIV-1-infected cells into the brain is the foundation of HIV-1-associated neurodegeneration; however, the outcome of HIV-1 CNS infection varies dramatically between individuals. Even before ART, disease progression to AIDS with and without neurocognitive impairment could take years. However, METH abuse exacerbates HIV-1-associated disease pathology, inducing changes that may last for decades even after METH is no longer abused (Cadet and Krasnova, 2007; Iudicello et al., 2014; Northrop and Yamamoto, 2015). HIVE, the most severe form of HAND, is pathologically characterized by inflammatory changes and accumulation of perivascular MP, formation of microglial nodules and multinucleated giant cells, astrogliosis, neuronal atrophy and death (Gendelman, 2005). With the effective use of ART helping to suppress disease progression, clinicians and researchers alike postulate that ANI and MND are stages of a similar disease process (Strazza et al., 2011). However, since HAND is often a comorbidity rather than the cause of death, HIV-1-associated neuropathology is often “non-specific,” leading many to search for other more subtle mechanisms of neurodegeneration (Gelman, 2015). Neuroinflammation remains a focus of intense study as inhibiting viral replication alone has slowed, but not halted, HAND progression.

### Neuroinflammation

The pro-inflammatory cascade leading to the disruption of the BBB and entry of HIV-1-infected leukocytes into CNS continues in the brain microenvironment. Resident microglia and perivascular MP perpetuate neuroinflammation, activating and or transmitting the infection to non-infected cells, including astroglia. As the resident immune cells, microglia are the primary HIV-infected cells in the brain mediating neuroinflammatory responses, by increasing cytokines, MMPs and cytotoxic factors (Ramesh et al., 2013). However, microglial activation and infection inevitably also lead to astrocyte activation and infection of a very small percentage of astrocytes with HIV. HIV infection in astrocytes is restricted to the extent that are capable of expressing viral proteins, including gp120, Tat and Nef, but not infectious virions (Messam and Major, 2000; Eugenin et al., 2011; Fitting et al., 2012; Li et al., 2015; Luo and He, 2015). Coculture experiments mimicking the interconnections between BMVEC and astroglia demonstrate that a small percentage (4.7%) of HIV-1-infected astrocytes can lead to endothelial apoptosis, dysregulation of lipoxygenase/cyclooxygenase (COX), calcium (Ca^2+^) channels and ATP receptor activation within astrocytes, significantly contributing to BBB disruption (Eugenin et al., 2011). Further, astrocytes exposed to HIV-1 proteins, along with those expressing them, have been shown to modulate to neuroinflammation through multiple regulatory pathways, summarized in Tables 1, 2.

**Table 1:** **Astroglial factors influencing neuronal survival and function**.

**Abbr**.	**Full length name**	**Receptor(s)**	**Additional function(s)/effect(s)**	**HIV/METH-associated references**	**Other CNS-associated references**
**INFLAMMATORY**					
AA	Arachidonic acid		Neurotoxic	Waschbisch et al., 2006; Samikkannu et al., 2011	
PGE_2_	Prostaglandin E2	PGE2R	Cerebral blood flow	Mollace et al., 1994; Falsig et al., 2004; Blanco et al., 2008; Samikkannu et al., 2011	Newman, 2015
C2, C3, C5	Complement components			Speth et al., 2001, 2002	Choi et al., 2014
CCL2, MCP-1	Monocyte chemoattractant protein-1	CCR2	Chemotaxis	Kutsch et al., 2000; Asensio et al., 2001; Roberts et al., 2010b; Mamik et al., 2011; Vartak-Sharma et al., 2014	Ransohoff et al., 1993; Smits et al., 2002; Strack et al., 2002; Croitoru-Lamoury et al., 2003; Ambrosini et al., 2005; Choi et al., 2014; Mayo et al., 2014
CCL3, MIP-1α	Macrophage inflammatory protein-1α	CCR1, 2, and 5	Chemotaxis		Smits et al., 2002; Ambrosini et al., 2005; Burkert et al., 2012
CCL4, MIP-1β	Macrophage inflammatory protein-1β	CCR3, CCR5	Chemotaxis	Choi et al., 2014	Smits et al., 2002; Ambrosini et al., 2005
CCL5, RANTES	Regulated on activation, normal T cell expressed and secreted	CCR1, 2, and 3	Chemotaxis	Choi et al., 2014; Liu et al., 2014a	Smits et al., 2002; Croitoru-Lamoury et al., 2003; Kim et al., 2004; Ambrosini et al., 2005; El-Hage et al., 2011
CCL7, MCP-3	Monocyte chemoattractant protein-3	CCR1 and 2	Chemotaxis	Renner et al., 2011	
CXCL1, Gro-α/β	Growth regulated oncogene-α/β	CXCR1 and 2	Chemotaxis		Coughlan et al., 2000; Wu et al., 2000; Lu et al., 2005; Choi et al., 2014
CXCL3, Gro-γ	Growth regulated oncogene-γ	CXCR2	Chemotaxis		Lu et al., 2005
CXCL5, ENA-78	Epithelial-derived neutrophil-activating peptide 78	CXCR2	Chemotaxis		Pang et al., 2001
CXCL6, GCP-2	Granulocyte chemotactic protein-2	CXCR2	Chemotaxis		Flynn et al., 2003; Lu et al., 2005
CXCL7, NAP-2	Neutrophil-activating protein-2	CXCR2	Chemotaxis		Lu et al., 2005
CXCL8, IL-8	Interleukin-8	CXCR1 and 2	Chemotaxis, Neuroprotection	Kutsch et al., 2000; Mamik et al., 2011	Xia et al., 1997; Puma et al., 2001; Croitoru-Lamoury et al., 2003; Flynn et al., 2003; Lu et al., 2005; Ashutosh et al., 2011; Choi et al., 2014
CXCL9, Mig	Monokine induced by interferon-γ	CXCR3	Chemotaxis, Dual-function	Asensio et al., 2001	Salmaggi et al., 2002; Croitoru-Lamoury et al., 2003; Flynn et al., 2003
CXCL10, IP-10	Gamma interferon inducible protein 1	CXCR3	Chemotaxis, Neurotoxic	Kutsch et al., 2000; Asensio et al., 2001; Mehla et al., 2012; Qin and Benveniste, 2012; Choi et al., 2014; Youn et al., 2014	Ransohoff et al., 1993; Salmaggi et al., 2002; Strack et al., 2002; Croitoru-Lamoury et al., 2003
CXCL11, I-TAC	Interferon-inducible T-cell α chemoattractant	CXCR3	Chemotaxis, Dual-function		Salmaggi et al., 2002; Croitoru-Lamoury et al., 2003; Hashioka et al., 2012
CXCL12, SDF-1α/β	Stromal cell-derived factor 1	CXCR4	Chemotaxis, Neurotoxic, HIV competitive inhibitor	Bleul et al., 1996; Oberlin et al., 1996; Bajetto et al., 1999; Kaul and Lipton, 1999; Vergote et al., 2006	Ambrosini et al., 2005; Shin et al., 2014
CXCL16		CXCR6	Chemotaxis		Ludwig et al., 2005
CCL20, MIP-3a	Macrophage inflammatory protein-3α	CCR6	Chemotaxis, Dual-function		Ambrosini et al., 2003, 2005; Zhou et al., 2011
CCL22, MDC	Macrophage-derived chemokine	CCR4	Chemotaxis, Dual-function	Youn et al., 2014	
CX3CL1	Fractalkine	CX3CR1	Chemotaxis		Yoshida et al., 2001
G-CSF	Granulocyte colony- stimulating factor	G-CSFR			Smits et al., 2002; Choi et al., 2014
GM-CSF, CSF 3	Granulocyte-macrophage colony-stimulating factor				Burkert et al., 2012; Choi et al., 2014; Mayo et al., 2014
IL-1α	Interleukin-1α	IL-1R			Smits et al., 2002
IL-1β	Interleukin-1β	IL-1R		Choi et al., 2014	Smits et al., 2002; Ambrosini et al., 2005; Burkert et al., 2012
IL-6	Interleukin-6	IL-6Rα chain (CD126) and gp130 (CD130)		Lee et al., 1993; Van der Meide and Schellekens, 1996; Falsig et al., 2004; Roberts et al., 2010b; El-Hage et al., 2011; Qin and Benveniste, 2012	Fiebich et al., 2001; Smits et al., 2002; Choi et al., 2014; Elain et al., 2014
IL-12	Interleukin-12	IL-12R-β1 and IL-12R-β2 complex			Constantinescu et al., 2005
IL-15	Interleukin-15	IL-2/15R (CD122)/CD132		Granado et al., 2011; Urrutia et al., 2014	Saikali et al., 2010
IL-16	Interleukin-16	CD4	Chemotaxis, anti-HIV	Maciaszek et al., 1997	Zhang et al., 2008
IL-18	Interleukin-18	IL-18R			Liu et al., 2014c
IL-19	Interleukin-19	IL-10R complex			Cooley et al., 2014; Nikfarjam et al., 2014
IL-23	Interleukin-23	IL-12R-β1 and IL-23 complex			Constantinescu et al., 1996, 2005; Parham et al., 2002
M-CSF	Macrophage colony stimulating factor	CSFR1			Smits et al., 2002
MIF	Macrophage migration inhibitory factor	CD74		Choi et al., 2014	
MMP-2	Matrix metalloproteinase-2			Dhar et al., 2006; Kou et al., 2009; Sbai et al., 2010; Peng et al., 2012	
MMP-3	Matrix metalloproteinase-3				Skuljec et al., 2011
MMP-9	Matrix metalloproteinase-9			Sbai et al., 2010; Yang et al., 2015	Kamat et al., 2014; Patel et al., 2015
MMP-12	Matrix metalloproteinase-12				Skuljec et al., 2011
TNF-α	Tumor necrosis factor-α	TNFR1/2		Lee et al., 1993; Van der Meide and Schellekens, 1996; El-Hage et al., 2011; Granado et al., 2011; Choi et al., 2014; Coelho-Santos et al., 2015	Smits et al., 2002; Ambrosini et al., 2005; Burkert et al., 2012
**NEUROTOXIC**					
H_2_O_2_	Hydrogen peroxide				Padovani-Claudio et al., 2006
NO	Nitric oxide			Mollace et al., 1994; Falsig et al., 2004; El-Hage et al., 2011; Castelli et al., 2014	Hu et al., 1998; Colombo et al., 2014; Mohsenzadegan et al., 2015
NOO^−^	Peroxynitrite			Muscoli et al., 2002; Castelli et al., 2014	
SDF 5-67	Stromal cell-derived factor 5-67	CXCR3		Vergote et al., 2006	
**HOMEOSTATIC**					
CCL19, MIP-3β	Macrophage inflammatory protein 3 β	CCR7			Pang et al., 2001; Columba-Cabezas et al., 2003
**ANTI-INFLAMMATORY**					
HO-1	Heme oxygenase-1		Anti-oxidant	Youn et al., 2014	
IL-10	Interleukin-10	IL-10R1 and 2 complex		Speth et al., 2000	Mohsenzadegan et al., 2015
IL-13	Interleukin-13	IL-4R and α IL-13-specific binding chain			Wynn, 2003; Burkert et al., 2012
IFN-α	Interferon-α	IFN-α/βR	Anti-viral	Zaritsky et al., 2012	
IFN-β	Interferon-β	IFN-α/βR	Anti-viral	Zaritsky et al., 2012	
TGF-β	Transforming growth factor-β	TGF-βR		Hori et al., 1999	Dhar et al., 2006; Endo et al., 2015; Weissberg et al., 2015
TIMP-1	Tissue inhibitor of metalloproteinases-1	β-1 integrin and CD63 complex	Neuroprotective	Sbai et al., 2010; Fields et al., 2011; Moore et al., 2011; Welser-Alves et al., 2011; Ashutosh et al., 2012	
TIMP-2	Tissue inhibitor of metalloproteinases-2		Pro-inflammatory	Sbai et al., 2010; Welser-Alves et al., 2011	Lee and Kim, 2014
**NEUROTROPHIC**					
BDNF	Brain-derived neurotrophic factor	Tropomyosin receptor kinase B (TrkB) and low affinity nerve growth factor receptor (LNGFR)		Saha et al., 2006	Patapoutian and Reichardt, 2001; Chen et al., 2005
GNDF	Glial derived neurotrophic factor	GDNF family receptor α 1 and 2	Astrotrophic		Chen et al., 2005; Yu et al., 2007; Penas et al., 2011
NGF	Nerve growth factor	TrkA			Chen et al., 2005
NT-3	Neurotrophin-3	TrkA, TrkB and LNGFR			Chen et al., 2005; Igelhorst et al., 2015

**Table 2:** **Astrocyte responses to HIV-1-relevant and METH stimuli**.

**Outcomes**	**HIV-1-relevant stimuli and METH**	**Regulation or mechanism**	**References**
**BBB PERMEABILITY**			
Increased CXCL10 expression, PBMC chemotactic activity	Virus, TNF-α	TNFR 1/2	van Marle et al., 2004
	Virus, IL-1β, TNF-α	CXCR3/CXCR4, MAPK, PKC	Mehla et al., 2012
	Tat_1−72_ treatment	p38 MAPK	Kutsch et al., 2000
Increased CCL2, CXCL8, CXCL10, ICAM-1 and vascular (V)CAM-1 expression	Tat treatment	MAPK, JNK, AP-1, NF-κB	Youn et al., 2014
Increased ICAM-1 expression leading to enhanced interactions with MP	gp120 treatment	PKC, TK, JAK2/STAT1α	Shrikant et al., 1996
Increased TNF-α expression leading to BBB impairment	METH	NF-κB pathway	Coelho-Santos et al., 2015
Dysregulation of TIMP-1: MMP Balance	Virus, IL-1β	CAATT-enhancer binding protein (C/EBP)-β, ERK 1/2, p38 MAPK	Suryadevara et al., 2003; Fields et al., 2011, 2013
	IL-1β	NF-κB, AP-1, PI3K, MAPK	Yang et al., 2015
**PRO-INFLAMMATORY**			
**Viral replication**			
Increased pro-viral replication (FIV)	METH	Viral entry or integration	Gavrilin et al., 2002
Increased pro-viral replication (HIV)	IFN-γ	STAT3 and Dickkopf-related protein 1, β-catenin	Li et al., 2011
**Cytokines and chemokine expression**			
Increased CCL2 production leading to regulation of IFN-α/β and TRAIL expression in MP	Virus (SIV)		Zaritsky et al., 2012
Increased C3 expression	Virus, Nef, gp41 treatment	Activation of adenylate cyclase, increased cAMP, IL-6/IL-1β responsive promoter elements and C/EBP-δ	Speth et al., 2002; Bruder et al., 2004
Increased C5, IL-1β, IL-1ra, TNF-α, CXCL10, CCL3, CCL5	IL-1β, TNF-α	NF-κB	Choi et al., 2014
Increased CCL20 expression	IL-1β, TNF-α		Ambrosini et al., 2003
Increased CCL5 expression	Nef treatment	Akt, p38 MAPK, NF-κB, C/EBP and AP-1	Liu et al., 2014a
	IL-1β, IFN-γ/β	IκBα, MAPKs, C/EBP-β, STAT1/2, interferon regulatory factor-1 (IRF-1)	Kim et al., 2004
Increased CCL2 expression	TNF-α	AEG-1 expression	Vartak-Sharma et al., 2014
Increased CCL7 expression	TNF-α		Renner et al., 2011
Increased CX3CL1 expression	TNF-α		Yoshida et al., 2001
Increased CCL2, CXCL8 and CD38 expression	HIV-1_YU−2_ expression	MAPK, ERK 1/2, NF-κB	Kou et al., 2009; Mamik et al., 2011
Increased CCL2, CXCL8 and CXCL10 expression	Tat treatment	MAPK, JNK, AP-1, NF-κB	Youn et al., 2014
Increased CCL2 and CXCL8 expression	Tat_1−72_ treatment	Mitogen-activated protein kinase kinase (MEK) ½	Kutsch et al., 2000
Increased CCL2, CXCL8 and IL-6 expression	VPR treatment		Ferrucci et al., 2013
Increased CXCL8 and CXCL10 expression	Virus, VPR treatment		Vivithanaporn et al., 2010
Increased CXCL8 and IL-6 expression	Tat expression	PI3K/Akt, p38 MAPK and NF-κB, p38δ, AP-1	Nookala and Kumar, 2014
	METH	mGluR5, Akt/PI3K, NF-κB	Shah et al., 2012b
Increased IL-6 and TNF-α expression	gp120 treatment		Van der Meide and Schellekens, 1996
Increased IL-6 expression	gp120 treatment	IκB kinase (IKK)β and NF-κB	Shah et al., 2011
**Eicosanoid expression and regulation**			
Increased TNF-α, IL-1β, leukotriene B4, leukotriene D4, lipoxin A4 and platelet-activating factor (PAF) expression	Virus (HIV-infected MP) coculture	Astrocyte cellular contact, AA-dependent	Genis et al., 1992
Increased prostaglandin EP3R expression	IL-1β	PKC, NF-κB	Waschbisch et al., 2006
Increased COX-2 expression	IL-1β	C/EBP-β	Fields and Ghorpade, 2012
Increased PGE2 expression	gp120 treatment	NO	Mollace et al., 1994
Increased COX-2, PGE2 and thromboxane A2 receptor expression	gp120 treatment (Clade B)		Samikkannu et al., 2011
Increased COX-2 and PGE2 expression	Tat treatment	NFAT, AP-1	Blanco et al., 2008
Increased IL-6, COX-2, PGE2 expression	IL-1β, TNF-α	p38 MAPK	Falsig et al., 2004
**OXIDATIVE STRESS**			
Increased ROS and NRF-2 anti-oxidant gene expression	gp120_BAL_ treatment		Reddy et al., 2012
Increased intracellular pH	gp120 treatment, IL-1β, TNF-α, IFN-γ	Na+/H+ exchange	Benos et al., 1994
Decreased DRD2 and DAT expression	gp120 treatment (Clade B), METH	CREB, CAMKII, CAMKIV	Samikkannu et al., 2015
Decreased ATP and GSH leading to increased ROS	VPR treatment		Ferrucci et al., 2013
Increased mitochondria depolarization	METH	ROS	Lau et al., 2000
Increased iNOS expression and NO levels	IL-1β, TNF-α, IFN-γ	p38 MAPK	Falsig et al., 2004
Increased iNOS expression	IFN-γ, IFN-β, LPS		Mohsenzadegan et al., 2015
**EXCITOTOXICITY AND NEURAL CELL TOXICITY**			
Decreased EAAT-2 expression and function	HIV-1_JR−FL_, IL-1β, METH	TAAR1, cAMP	Cisneros and Ghorpade, 2012, 2014
	IL-1β	AEG-1	Vartak-Sharma et al., 2014
Decreased NMDA receptor expression and glutamine levels	gp120 treatment (Clade B)		Samikkannu et al., 2011
Increased CXCL10 expression leading to neuronal toxicity	Virus, Nef_YU−2_ expression, TNF-α	TNFR1/2	van Marle et al., 2004
	Virus, IL-1β, TNF-α	CXCR3/CXCR4, MAPK, PKC	Mehla et al., 2012
	IL-1β, α-synuclein		Tousi et al., 2012
Increased astrocyte apoptosis	gp120 treatment		Van der Meide and Schellekens, 1996
	IL-1β, TNF-α, IFN-γ	CD95, caspase 8, FADD	Falsig et al., 2004; Gardner et al., 2006
**ASTROGLIAL PHYSIOLOGICAL FUNCTIONS**			
**Neurotrophic**			
Increased BDNF expression	TNF-α	NF-αB, C/EBP-β with ERK MAPK	Saha et al., 2006
	Glutamate	PLC, IP_3_, internal stores of Ca^2+^	Jean et al., 2008
**Anti-inflammatory**			
Reduced eicosanoids, platelet-activating factor (PAF) and TNF-α	Virus (HIV-infected MP) coculture	Astrocyte cellular contact	Nottet et al., 1995
Decreased viral replication in MP	Virus (M tropic)	Latent TGF-β expression in astrocytes	da Cunha et al., 1995; Hori et al., 1999
Increased TGF-β1	Tat treatment and expression		Wahl et al., 1991; Cupp et al., 1993; Rasty et al., 1996; Thatikunta et al., 1997
Decreased IL-1β-induced TIMP-1 and MMP-2 expression, increased ECM levels	IL-1β	TGF-β 1/2	Wyss-Coray et al., 1995; Dhar et al., 2006
Increased IL-10 expression	gp41 treatment	adenylate cyclase, cAMP	Speth et al., 2000
	IFN-γ/β, LPS		Mohsenzadegan et al., 2015

Pro-inflammatory molecules also propagate inflammation by the spread of reactive gliosis and affect neuronal function and survival by direct and indirect mechanisms. In the healthy nervous system, cytokines and chemokines are neuromodulators, regulating neurodevelopment, neuroinflammation, and synaptic transmission. They are fundamental to the brain's proper immune function, serving to maintain immune surveillance, facilitate leukocyte traffic, and recruit other inflammatory factors (Chui and Dorovini-Zis, 2010). However, during neuroinflammation associated with both HIV-1 infection and METH exposure, activated glia mediate neuronal injury and death through neurotoxic signaling, generation of ROS, altered cellular metabolism, neurotransmission and cerebral blood flow, among others (Lau et al., 2000; Abdul Muneer et al., 2011; Hoefer et al., 2015). In such, reactive glia, infected or not, participate in the disruption of the BBB leading to infiltration of HIV-1-infected cells into the CNS and continuation of neuroinflammation in the brain. The specific contributions and regulation of these cytokines, chemokines and bioactive molecules in reactive astrocytes and other cells during HIV-1 and METH comorbidity are summarized in Tables 1, 2 and will be discussed in more detail below.

### Oxidative stress

ROS participate in signaling and metabolic pathways during physiological conditions (Ray et al., 2012). During homeostasis, anti-oxidant enzymes, including super oxide dismutase (SOD), glutathione peroxidase (GP), glutathione (GSH) and catalase (CAT), tightly regulate and neutralize reactive molecules such as superoxide, hydrogen peroxide and hydrogen radicals. Excessive ROS induced by a variety of mechanisms, including inflammatory cytokines, mitochondrial respiration, ischemia and infection, are implicated in aging, cardiovascular disease, diabetes, stroke and neurodegeneration (Cobb and Cole, 2015; Raz et al., 2015; Salisbury and Bronas, 2015). Reactive nitrogen species (RNS) also contribute the oxidative environment (Torre et al., 2002). Inducible NO synthase (iNOS) generates NO, which can interact with ROS to form peroxynitrite, a highly RNS (Pacher et al., 2007). Low levels of oxidative stress activate anti-oxidant machinery, initiate anti-microbial responses in immune cells and activate endothelial cells (Ma et al., 2014; Molteni et al., 2014; Salisbury and Bronas, 2015). Unchecked oxidative and nitrosative modifications to cellular components, such as the mitochondria, often augment oxidative stress and induce apoptosis (Cossarizza et al., 2002; Jou, 2008; Indo et al., 2015). Oxidative stress in the HIV-infected brain, through both the early effects of viral proteins and the later effects on mitochondrial integrity, are well established (Valcour and Shiramizu, 2004; Ozdener, 2005; Banerjee et al., 2010; Uzasci et al., 2013). Low ROS levels can promote viral replication and can be induced by viral virulence factors (Molteni et al., 2014). HIV-1 gp120, Tat and viral protein R (Vpr) induce ROS in neural cells, including astrocytes (Ferrucci et al., 2013; Shah et al., 2013). In addition to the direct generation of oxidative stress by HIV-1, antiretroviral therapies, particularly dideoxynucleotide reverse transcriptase inhibitors (NTRI), alter mitochondrial function and exacerbate oxidative damage in peripheral and central nervous systems (Lewis, 2003; Akay et al., 2014). During HIV disease progression, increased oxidative stress is accompanied by deficiencies in anti-oxidant enzymes, such as GP, GSH and SOD (Gil et al., 2003; Sundaram et al., 2008; Pang et al., 2013; Gill et al., 2014; Samikkannu et al., 2014). Astrocytes treated with indinavir or nelfinavir export GSH into the culture supernatant, indicating brain GSH homeostasis may also be dysregulated by HIV-1 protease inhibitors in astrocytes (Brandmann et al., 2012).

Relative oxidative stress also differs between HIV clades and may contribute to the neuropathogenesis of clade B as compared to clade C (Samikkannu et al., 2014). Clade B virus induced production of ROS, coupled with reduced expression of GSH synthase, GP, SOD and CAT, in monocyte derived dendritic cells and neuroblastoma cells compared to clade C virus (Samikkannu et al., 2014). The expression of detoxifying enzymes heme oxygenase (HO)-1 and NAD(P)H dehydrogenase increased in HIV-1 gp120-treated astrocytes (Reddy et al., 2012). However, HO-1 levels are decreased in the brain of HIV-1-infected individuals and correlate with increased cognitive dysfunction (Gill et al., 2014). An inability for astroglia and other neural cells to maintain anti-oxidant responses may implicate anti-oxidant exhaustion in the chronic neurodegenerative disease process.

Contributions of oxidative stress to METH-mediated neurotoxicity are also well accepted. Overexpression of various SODs or knockout (KO) of neuronal NOS, abrogate striatal depletion of dopamine and serotonin (Cadet et al., 1994; Hirata et al., 1995; Itzhak et al., 2000; Maragos et al., 2000). Regional differences in the anti-oxidant capacity of brain regions of HIV-1 transgenic rats exposed to METH show independent and combined effects on induction of oxidative stress (Pang et al., 2013). Coexposure to both HIV and METH increases oxidative stress and apoptosis in the brain, which is associated with neurological impairment (Banerjee et al., 2010; Ferrucci et al., 2013; Shah et al., 2013). Administration of N-acetylcysteine amide (NACA), a thiol anti-oxidant, protected the BBB from oxidative stress-mediated damage in HIV-1 gp120-, Tat- and METH-treated mice (Banerjee et al., 2010). Together these data support the importance of oxidative stress in HIV- and METH-mediated neurodegeneration.

### Excitotoxicity

Excitotoxicity is a direct result of abnormal regulation of glutamate concentrations in the synapse. As a common neurotoxic mechanism, excitotoxicity is implicated in many neurodegenerative conditions including HAND and METH abuse (Erdmann et al., 2006; Jaiswal et al., 2009; Vázquez-Santiago et al., 2014). During physiologic glutamatergic signaling, glutamate interacts with its receptors, N-methyl-D-aspartate receptor (NMDA) and α-amino-3-hydroxy-5-methyl-4-isoxazolepropionic acid receptor (AMPA), to induce a Ca^2+^ influx and potentiation of the excitatory signal. Excitatory amino acid transporters (EAAT)-2 on adjacent astrocyte processes quickly clear glutamate from the synapse to end post-synaptic neuron excitation (Camacho and Massieu, 2006). Pathologically elevated levels of glutamate trigger high levels of intracellular Ca^2+^ ([Ca^2+^]_i_) and activate a variety of enzymes, including phospholipases, endonucleases and proteases. Opening of mitochondrial permeability transition pores, upon uptake of excessive Ca^2+^, releases ROS and pro-apoptotic compounds (Manev et al., 1989; Ankarcrona et al., 1995; Stavrovskaya and Kristal, 2005).

Plasma and CSF glutamate levels are elevated in HAD patients (Ferrarese et al., 2001; Espey et al., 2002). HIV-1 infected macrophages and microglia convert glutamine to glutamate through the release of glutaminase from compromised mitochondria (Erdmann et al., 2009). In such MP and microglia increase extracellular glutamate levels by secreting both glutamate and glutaminase (Huang et al., 2011). HIV-1 Tat also prolongs glutamate triggered Ca^2+^ influx by inducing the phosphorylation of NMDA receptors, leading to enhanced cell death (Haughey et al., 2001). In human brain tissues, EAAT-2 expression was decreased in HIV+ individuals, with and without HIVE. EAAT-2 expression correlated with areas of diffuse microglial reactivity, indicating that microglial activation contributes to astrocyte dysfunction (Xing et al., 2009). Astrocytes are responsible for clearing ~90% of extracellular glutamate from the synapse. HIV-1 decreases EAAT-2 expression in cultured human astrocytes (Wang et al., 2003) and glutamate clearance is impaired by inflammation (Cisneros and Ghorpade, 2012, 2014).

METH alters the expression, composition and function of NMDA and AMPA receptors in the brain. Receptor levels increased with both acute and chronic models of METH administration and isoforms of Ca^2+^-impermeable receptors increased, suggesting a mechanism to counteract METH-induced excitotoxicity (Simões et al., 2008). The frequency of spontaneous and miniature excitatory postsynaptic currents increases at low METH doses and begin decreasing at higher doses (Zhang et al., 2014). Extracellular glutamate concentrations remain elevated, even after dopamine levels return to physiological levels (Mark et al., 2007). METH downregulates astrocyte EAAT-2 expression through trace amine associated receptor (TAAR)1, which is also associated with regulation of dopamine transporters in neurons (Cisneros and Ghorpade, 2014). EAAT-2 dysregulation in reactive astrocytes likely contributes to HIV- and METH-mediated excitotoxicity.

## Gliosis

Although infiltration of peripheral immune cells often leads to significant neural damage, leukocyte infiltration is not always associated with neurotoxicity (Boztug et al., 2002; Trifilo and Lane, 2003; Clark et al., 2011). In such, the resident glial cells, microglia and astroglia, are implicated as central players in the inflammatory responses associated with neurodegeneration. The term gliosis refers to a non-specific reactive change in glial cells in response to damage, disease or infection in the CNS. Reactive glia are often identified in brain tissue by morphological changes, including increased size, elongation of processes and increased reactivity with cellular markers. Morphological changes are indicative of altered glial function. The primary goal of gliosis is to restore brain homoeostasis by providing trophic support, tissue repair and containment of the affected region. As discussed above, reactive glia secrete many neuroactive substances capable of injuring neural cells, dependent upon the type, severity and duration of insult. Ultimately, the balance between the beneficial and detrimental effects of gliosis determines the outcome in the CNS.

### Microglia

Microglia make up between 10 and 15% of neural glia and are accountable for the innate immune response in the brain (Lawson et al., 1992; Verkhratskiǐ and Butt, 2013; Elmore et al., 2014). The homeostatic functions of microglia tend to go unnoticed in the brain, even though they play an active role in embryonic brain development and clear neuronal or glial debris, while surveying their environment for threat and injury (Beyer et al., 2000; Casano and Peri, 2015). When injury or infection is detected, microglia undergo dramatic morphologic alterations, shifting from resting ramified cell into an activated amoeboid phenotype, and transforming into a more classically functioning immune cell (Burdo et al., 2013; Tavazzi et al., 2014). Activated microglia upregulate surface receptors, including major histocompatibility complex molecules, leading to secretion of factors that influence neuronal survival and a chronic neuroinflammatory state (Streit, 2000; Block and Hong, 2005). Reactive microgliosis is associated with the pathogenesis of many common types of neurodegeneration, including HAND (da Fonseca et al., 2014; Pasqualetti et al., 2015).

### Astroglia

Despite the abundance of astrocytes in the brain, their pro-inflammatory functions have been less prominent than their microglial counterparts and continues to evolve (Ghorpade et al., 2003; Block and Hong, 2005; Ladeby et al., 2005; Ashutosh et al., 2011; Fields and Ghorpade, 2012; Van der Meide and Schellekens, 1996; Vartak-Sharma et al., 2014; Abudara et al., 2015). However, astrocytes play critical physiological roles in the brain, providing glia-neuron contact, ionic homeostasis, neurotransmitter buffering, secretion of neurotrophic factors and serve as integral members of the BBB (Van der Meide and Schellekens, 1996). Therefore, alterations in astroglial activities during reactive astrogliosis directly affect neuronal function and survival during CNS insult and infection (Abudara et al., 2015). Further, astrocyte dysfunction during neuroinflammation, injury and disease is amplified by the sheer number of cellular interactions in which each cell participates, stretching from BMVECs at the BBB to individual neurons and synapses (Giaume et al., 1997; Butt, 2011; Li et al., 2014; Muñoz et al., 2015). Astrocytes express glutamatergic, GABAergic, adrenergic, purinergic, serotonergic, muscarinic, and peptidergic receptors (reviewed in Porter and McCarthy, 1997). Thus, activated astrocytes respond to various neurotransmitters and release a variety of neuroactive molecules including glutamate, ATP, NO, and prostaglandins to influence neuronal function (Haydon, 2001; Table 1). Astrocytes are highly susceptible to cytokine and HIV-1 signaling as they express receptors (R) for both (IL-1R, TNFR1/2 and CXCR4, among others; Table 2). Activated astrocytes secrete various cytokines and chemokines regulating leukocyte traffic into the brain (Peng et al., 2006; Ramesh et al., 2013; Nookala and Kumar, 2014). However, as most astrocyte responses are complex, astrocytes also mitigate inflammation with the secretion of anti-inflammatory and neurotrophic molecules (Table 1; Hauwel et al., 2005; Ashutosh et al., 2011, 2012; Cekanaviciute et al., 2014). In addition, astrocytes are the primary cells mediating glial scar formation during brain injury such as stroke and parasitic infections associated with AIDS (Kielian, 2004; Voskuhl et al., 2009). During glial scarring astrocytes migrate and replicate to encapsulate injury, which in turn impairs repair and neurite regrowth (Cregg et al., 2014; Hermann et al., 2014; Raposo and Schwartz, 2014).

As a pathological hallmark of HIVE, reactive astrogliosis is apparent in mouse and human HIV+ brain tissues. Astrogliosis is often visualized histologically by increased glial fibrillary acidic protein (GFAP) staining, near areas of active HIV-1 replication in multinucleated giant cells and microglial nodules (Reviewed in Sabri et al., 2003; Tavazzi et al., 2014). Astrocyte activation is also prevalent at perivascular locations, even in the absence of HIV+ perivascular MP, implicating astrocyte dysregulation of the BBB as a mechanism of neuroinflammation (Tavazzi et al., 2014). Several cytokines and other soluble MP activation factors implicated in HIVE pathogenesis, including TNF-α, IL-1β, NO and glutamate are reported to upregulate GFAP expression in astrocytes (Zhang et al., 2000; John et al., 2003; Brahmachari et al., 2006). HIV-1-Tat-induced increases in GFAP expression are regulated by the sequential activation of early growth response protein 1 (Egr-1) and p300 through a signal transducer and activator of transcription 3 (STAT3)-dependent mechanism (Zou et al., 2010; Fan et al., 2015). A similar mechanism is seen in METH-induced astrogliosis, which activates the Janus kinase 2 (JAK2)/signal transducer and STAT3 signaling cascade (Hebert and O'Callaghan, 2000; Robson et al., 2014). However, induction of astrogliosis in METH abuse studies is inconsistently increased across brain regions, METH dosing strategies and time points (Ernst et al., 2000; Kita et al., 2003, 2009; Moszczynska et al., 2004; Cadet and Krasnova, 2009; Krasnova and Cadet, 2009). In post-mortem brains of chronic METH abuse or overdose, the contribution of astrocytes to the neurodegenerative disease process is often reduced to alterations in GFAP expression or reactivity (Granado et al., 2011; Shah et al., 2013; Silva et al., 2014; Tong et al., 2014). While changes in GFAP expression are representative of astrocyte activation, they are not indicative measures of changes in the multitude of astrocyte functions capable of influencing neuron function and survival, which have primarily been investigated *in vitro*. Concurrently, some investigators examine astrocyte functions through behavioral testing and neuronal functional assays along side gene expression studies to evaluate changes in proteins implicated in astrocyte-mediated neurodegeneration (Roberts et al., 2010a; Liu et al., 2014b; Hoefer et al., 2015).

## METH abuse: implications for astrocytes as viral reservoirs

HIV-1 can invade the CNS early during infection, primarily infecting infiltrating monocytes and resident microglia, along with a small proportion of astroglia. HIV-1 then integrates with the host cell genome as a provirus, leading to both active and latent infection. During active HIV-1 infection in permissive cells, budding of infectious virions ensues. However, in non-permissive cells such as astrocytes, active HIV-1 infection is restricted to expression of viral proteins, which are incapable of maturing into infectious particles (Messam and Major, 2000; Eugenin et al., 2011; Fitting et al., 2012; Li et al., 2015; Luo and He, 2015). Viral replication is limited in astrocytes at various steps of the virus life cycle including virus entry, reverse transcription, transport and translation of viral RNA, and maturation of progeny virions (reviewed in Messam and Major, 2000; Gorry et al., 2003). Other studies suggest that if restrictions on viral entry into astrocytes are bypassed, the intracellular environment may be conducive to productive viral infection (Canki et al., 2001; Chauhan, 2014).

Astrocytes lack the CD4 coreceptor that interacts with gp120 coat protein, restricting the proportion of astrocytes ultimately infected with HIV-1 (Harouse et al., 1989). In early studies, human embryonic astrocytes were found to express CCR5 and CXCR4 transcripts; however, neither R5 nor X4 tropic viruses could directly infect pure astrocyte cultures (Boutet et al., 2001). Recently, viral entry of fluorescently labeled viral RNA in HIV-1 NL4-3 virions was visualized in human astrocytes (Xu et al., 2015) and by mannose receptor-mediated endocytosis (Liu et al., 2004). In addition, astrocytes are susceptible to direct viral transfer of either R5 or X4 tropic viruses by cell-to-cell contact with infected T lymphocytes (Li et al., 2015; Luo and He, 2015). Viral transfer though the formation of virological synapses between astrocyte and lymphocyte filopodia can be blocked by CXCR4 antibodies and antagonists (Li et al., 2015). Further, astrocytes need not secrete mature virions to directly infect neighboring cells (Luo and He, 2015) and can “trans-infect” T lymphocytes by protecting exogenous HIV-1 particles in CD81-lined vesicles (Gray et al., 2014). Thus, if infected astrocytes are capable of directly propagating CNS HIV-1 infection, the elimination of latent astrocyte infection needs to be aggressively studied as HIV-1 replication may be reactivated by inflammation and drug abuse (Gavrilin et al., 2002; Carroll-Anzinger et al., 2007; Li et al., 2011; Chauhan, 2015).

In the brains of HIV-1-infected individuals with METH dependence, epigenetic changes were associated with increased global DNA methylation as compared to the brains of HIV-1+ individuals without METH use. METH exposure led to differential methylation in genes connected to neurodegeneration, oxidative phosphorylation, dopamine metabolism and transport (Desplats et al., 2014). Differential regulation of gene expression in microglia and astrocytes during METH and HIV comorbidity may induce viral replication and expression of pro-inflammatory mediators to contribute to neurodegeneration. METH enhances viral replication in macrophages and may upregulate or downregulate infection in T cells (Liang et al., 2008; Wang et al., 2012; Mantri et al., 2014). METH activates transcription of proviral DNA in latently HIV-1-infected human microglial cells, leading to activation of the NF-κB signaling pathway (Wires et al., 2012). Feline immunodeficiency virus (FIV), a lentivirus related to HIV-1, leads to astrogliosis and microgliosis. METH has been shown to increase cell-associated FIV replication in feline astrocytes and cell lines (Phillips et al., 2000; Gavrilin et al., 2002). Reactivation of viral expression in latently infected astrocytes could contribute to either increased neuroinflammation and toxicity or the elimination of viral reservoirs by viral cytopathic effects and lysis by effector cells. During METH, adherence to ART is decreased and the immune system is depressed (Reback et al., 2003; In et al., 2005), tipping the balance toward increased HIV-1- and METH-associated neurodegeneration. A quick, wide-spread activation of latently infected cells, coupled with effective ART delivery to counter the spread of infection, may lead to the clearance of HIV-1-infected neural cells (Díaz et al., 2015). However, the implications of widespread elimination of infected astrocytes and other latently infected cells on neural function are unknown; the results of which may favor strategies for maintaining a latent CNS infection, rather than radical activation and elimination. (reviewed by Brew et al., 2013; Churchill and Nath, 2013).

## Astrocyte interactions with HIV-1 virions, proteins, and METH

In astrocytes, expression of and exposure to virus, HIV-1 proteins, such as gp120, Tat, Nef, or Vpr, and HIV-1-relevant cytokines induce a host of factors that influence neuronal survival and function (Table 2). Both HIV-1 and METH alter astrocyte expression of inflammatory mediators, neurotransmitter receptors and transporters, which in turn alter the brain microenvironment, leading directly and indirectly to neuronal dysfunction or death. HIV-1-relevant cytokines also regulate astrocyte cytotoxicity, function and glia-neuron crosstalk during HIV-1 infection and METH abuse.

Astrocytes harboring HIV secrete various viral proteins, including gp120, Tat, Vpr and p24, the capsid protein. In some models of latent astrocyte infection, viral expression has been reactivated by pro-inflammatory cytokines such as TNF-α and IFN-γ or PKC agonists (Carroll-Anzinger et al., 2007; Li et al., 2011; Chauhan, 2015). Expression of viral proteins activates both the infected cell and those in the vicinity by altering astrocyte physiological functions and secretion of factors that recruit immune cells and influence neuronal survival and function (Table 2). Direct interaction between astrocytes and HIV-infected MP reduces MP activation, but ultimately increases arachidonic acid (AA)-mediated eicosanoid, IL-1β and TNF-α levels (Genis et al., 1992; Nottet et al., 1995). SIV/HIV-treated and HIV-genome expressing astrocytes upregulate complement and chemokine expression, leading to increased PBMC infiltration (Speth et al., 2002; Bruder et al., 2004; Vivithanaporn et al., 2010; Zaritsky et al., 2012). HIV-1 also downregulates astrocyte EAAT-2 expression and function, contributing to excitotoxicity (Cisneros and Ghorpade, 2012, 2014).

### HIV-1 gp120

As a viral coat protein, HIV-1 gp120 interacts with CCR5 and CXCR4 coreceptors on target cells leading to intracellular signaling and virion fusion with the cell. CXCR4 renders astrocytes susceptible to activation by HIV-1 *via* gp120-coated virus and secreted gp120. Astrocytes exposed to gp120 undergo apoptosis, while also inducing neuronal apoptosis. In astrocytes HIV-1 gp120 upregulates pro-inflammatory cytokines, adhesion proteins, and chemokines that mediate lymphocyte recruitment and extravasation (Table 2; Shrikant et al., 1996; Van der Meide and Schellekens, 1996; Kaul and Lipton, 1999).

Differences in astrocyte responses to clade B vs. clade C gp120 may contribute to increased neurodegeneration associated with clade B viruses. Clade B gp120 differentially increases COX-2-mediated AA responses in astrocytes, leading to downregulation of NMDA receptor expression and increasing PGE2 (Samikkannu et al., 2011). Bioactive molecules, such as METH, NO and PGE2, regulate the pro-inflammatory environment, cerebral blood flow and glucose uptake in the brain, contributing to HIV- and METH-associated neurodegeneration (Mollace et al., 1994; Falsig et al., 2004; Blanco et al., 2008; Abdul Muneer et al., 2011; Samikkannu et al., 2011). Further, during cotreatment with METH, clade B gp120 significantly decreased astrocyte expression of both dopamine receptor D2 and dopamine active transporter (DAT) as compared to METH alone or clade C gp120 (Samikkannu et al., 2015). Downregulation of dopamine receptors and transporters could impair astrocyte responses to increased synaptic dopamine levels, leading to reduced dopamine clearance and dopamine-mediated neurotoxicity through the generation of reactive dopamine quinones and oxygen/nitrogen species (Mollace et al., 1994; LaVoie and Hastings, 1999; Lau et al., 2000; Falsig et al., 2004; Guillot et al., 2008; Miyazaki et al., 2011; Castelli et al., 2014).

Behavioral testing in transgenic mice expressing HIV-1 gp120, under the control of the GFAP promoter, with and without METH administration, showed impaired learning and memory and increased disinhibition even after months of METH abstinence (Hoefer et al., 2015). Both METH and gp120 alone lead to loss of dendrites and presynaptic terminals, along with reduced long-term potentiation, which is associated with learning and memory. Further, post-tetanic potentiation, a measure of synaptic plasticity, was also decreased in METH-treated, gp120-transgenic mice (Hoefer et al., 2015).

### HIV-1 Tat

As its name suggests, HIV-1 Tat activates transcription of HIV-1 genes during viral infection. However, Tat also regulates expression of cellular genes as a transcription factor and by altering signaling within the cell. Similar to gp120-treated astrocytes, Tat-exposed/expressing astrocytes have increased expression of various cytokines, chemokines, prostaglandins, adhesion protein expression, neurotransmitter receptors and transporters, and ROS (Table 2). HIV-1 Tat-mediated neurotoxicity is exacerbated by METH cotreatment, leading to increased autophagy, mitochondrial damage and oxidative stress in neuronal cell lines and mouse astrocytes (Lau et al., 2000; Langford et al., 2004; Cai and Cadet, 2008; Qi et al., 2011). Further, rodents cotreated with HIV-1 Tat and METH showed increased astroglial activation and synergistic cytokine expression (including TNF-α and IL-1β), oxidative stress, coupled with striatal neurotoxicity and degeneration of neuronal dopamine terminals (Flora et al., 2003; Theodore et al., 2006b; Liu et al., 2014b). METH and Tat synergistically reduce dopamine levels and DAT expression, contributing to sustained behavioral impairment (Cass et al., 2003; Liu et al., 2014b). In double TNFR1/2 KO mice, dopamine levels were significantly higher than WT treated with Tat and METH, indicating the involvement of TNF-α and inflammation in neurodegenerative mechanisms (Theodore et al., 2006b).

### HIV-1 Nef

As a virulence factor HIV-1 Nef is expressed early during the viral life cycle and ensures a persistent state of infection, while promoting T-cell activation. Whereas, HIV-1 gp120 and Tat exhibit direct astroglial and neuronal toxicity, HIV-1 Nef has been shown to induce indirect neurotoxicity through upregulation of astroglial CXCL10. Astrocytes expressing HIV-1_YU−2_ Nef protein showed increased CXCL10 expression. CXCL10 mediated neurotoxicity through interaction with neuronal CXCR3 (van Marle et al., 2004). CXCL10 levels are also increased in HAD brains where it localizes primarily to astrocytes and is a prognostic marker for hepatitis C virus (HCV) and HIV/HCV coinfection (van Marle et al., 2004; Falconer et al., 2010; Vivithanaporn et al., 2010). CXCL10 is also known as IFN-γ induced protein (IP)-10. As a chemokine, CXCL10 recruits MP and T cells and promotes cell adhesion to BMVEC (Dufour et al., 2002). Astrocyte exposure to Tat also increases CXCL10 and expression of adhesion proteins such as ICAM-1 and VCAM-1, which together lead to increased trafficking of T cells into the brain (Kutsch et al., 2000; Dufour et al., 2002; Youn et al., 2014). Further, astrocyte CXCL10, from gp41-treated astrocytes, leads to increased CCR5 expression by MP, increasing their susceptibility to HIV-1 infection (Speth et al., 2000).

Transgenic mice, expressing HIV-1 Nef in microglia and macrophages, showed increased CCL2 expression, decreased anti-viral IFN-α expression and disruption of striatal dopaminergic transmission. Monoamine oxidase activity and DAT expression in the striatum were significantly lower than non-transgenic mice. Astroglial activation was not evaluated. The Nef-expressing mice demonstrated hyperactive behaviors, which are observed in mania and other psychiatric comorbidities among HIV-infected individuals (Sherbourne et al., 2000; Acharjee et al., 2014). This suggests that HIV-1 Nef could also regulate the dopaminergic system during HIV CNS infection and METH abuse.

### HIV-1-relevant cytokines

In addition to direct astrocyte activation by binding of HIV-1 gp120 to CXCR4 or viral endocytosis (Liu et al., 2004; Fitting et al., 2012; Chauhan et al., 2014), astrocytes may also become indirectly stimulated by HIV-infected and activated microglia and MP (Tavazzi et al., 2014). Infiltrating monocytes and T helper cells secrete classical inflammatory cytokines into the brain microenvironment during HIV-1 CNS infection, leading to astrocyte activation and increased neuroinflammatory responses. With prolonged exposure to HIV-relevant neuroinflammation, astrocytic neuroprotective and homeostatic functions become exhausted, leading to insufficient support of neuronal function and survival (Gardner and Ghorpade, 2003; Suryadevara et al., 2003; Cisneros and Ghorpade, 2012). Alternatively, chronic neurodegeneration can also prime astrocytes for exaggerated pro-inflammatory responses (Hennessy et al., 2015). Concurrent and long-term exposure of astrocytes to HIV, pro-inflammatory cytokines and METH can exacerbate astrocyte activation and exhaustion to accelerate the neurodegenerative process (Cisneros and Ghorpade, 2012, 2014; Shah et al., 2012a).

As prototypical mediators of neuroinflammation, IL-1β and TNF-α are primarily expressed in the CNS by activated and HIV-1-infected microglia and infiltrating MP (Mrak and Griffin, 1997; Witwer et al., 2009). Astrocytes are highly sensitive to IL-1β-activation, as they possess an IL-1β autocrine loop, which perpetuates astrogliosis in a self-renewing manner during chronic neuroinflammation, neurodegeneration and HAND (Mrak and Griffin, 1997; Moynagh, 2005). TNF-α, in conjunction with HIV, is a key regulator of astroglia-microglia crosstalk during neurodegeneration and can directly target neurons through TNFR1/2 and increased oxidative stress leading to apoptosis (Shi et al., 1998; Viviani et al., 1998; Ryan et al., 2004; Batlle et al., 2015). TNF-α regulates astrogliosis and impairs astrocyte function during HIV-1 and METH exposure (Nomura et al., 2006; Gonçalves et al., 2008; Vartak-Sharma et al., 2014; Coelho-Santos et al., 2015). Upon activation, astrocytes convert from flat, polygonal cells to small, contracted, highly branched cells, with intense GFAP and vimentin reactivity (Liu et al., 1994). IL-6, CCL2 and CXCL8 are upregulated in astroglia in response to HIV-relevant stimuli, including IL-1β activation and expression of viral proteins, and are increased in the plasma and brain during HIV-1 infection (Linder and Gagel, 1968; Cota et al., 2000; Witwer et al., 2009; Jing et al., 2010; Mamik et al., 2011; Shah et al., 2011; Mamik and Ghorpade, 2012; Zaritsky et al., 2012; Nookala and Kumar, 2014; French et al., 2015). These, and other cytokines, signal to peripheral and tissue immune cells, recruiting them to the site of neuroinflammation, inducing maturation and activating their effector functions.

Cytokines and chemokines can have alternate, indirect functions on non-immune cells in the brain, leading to both neuroprotective and neurotoxic outcomes. For example, CXCL8 has neuroinflammatory and neuroprotective effects in the CNS, as CXCL8 enhances viral replication in monocytes and microglia, while protecting neurons from apoptosis (Ashutosh et al., 2011; Mamik and Ghorpade, 2014). Further, METH exposure induces CXCL8 expression in SVG astrocytes. Regulation of CXCL8 expression through metabotropic glutamate receptor 5 (mGlutR5) implicates glutamate dysregulation in METH-induced neuroinflammation (Shah et al., 2012a,b). A more complex example involves the upregulation of CXCL12, MMP-2 and stromal cell derived factor (SDF) 5-67 during HIV-1 CNS infection (Vergote et al., 2006). Infected or gp120-treated MP regulate astrogliosis by secreting CXCL12 and IL-1β. In response, activated astrocytes secrete both CXCL12 and MMP-2 (Bajetto et al., 2001; Rostasy et al., 2003; Okamoto et al., 2005; Peng et al., 2006). These factors share a unique interaction where in MMP-2, an enzyme normally involved in the degradation of the extracellular matrix, cleaves CXCL12 to generate SDF 5-67. As a cytotoxic fragment SDF 5-67 induces neurotoxicity and upregulates IL-1β, TNF-α, indoleamine 2′,3′ dioxygenase (IDO) and IL-10 in activated astrocytes (Vergote et al., 2006). Alternately, CXCL12 impairs HIV-1 infection by CXCR4 tropic virus by competitively binding CXCR4 and blocking interactions with gp120 (Bleul et al., 1996; Oberlin et al., 1996; Amara et al., 1997; Kaul and Lipton, 1999).

Downregulation of astrocyte EAAT-2 expression and function by pro-inflammatory cytokines contributes to HIV-1- and METH-associated excitotoxicity. During chronic HIV-1 infection, MP/microglia glutamate secretion increases and HIV-1 Tat sensitizes neurons to glutamate-mediated excitotoxicity (Haughey et al., 2001; Erdmann et al., 2009; Huang et al., 2011). EAAT-2 is the primary transporter for glutamate uptake in astrocytes. Astrocyte activation by IL-1β or TNF-α decreases both the expression and function of EAAT-2; effects that are exacerbated by HIV-1 or METH cotreatment (Fine et al., 1996; Cisneros and Ghorpade, 2012). Gene expression, post-translational modifications and protein targeting or trafficking regulate EAAT-2 activity (reviewed in Takahashi et al., 2015). The EAAT-2 promoter contains multiple NF-κB elements and a CREB binding element (Su et al., 2003; Allritz et al., 2010). Both signaling cascades are activated in astrocytes during HIV-1, METH and neuroinflammation (Mamik et al., 2011; Samikkannu et al., 2015). Astrocyte elevated gene-1, first identified as an HIV-1 and TNF-α–inducible gene, contributes to IL-1β/TNF-α/HIV-1-mediated downregulation of EAAT-2 through direct interactions with NF-κB (Kang et al., 2005; Vartak-Sharma et al., 2014). The multifaceted mechanisms regulating EAAT-2 expression and function remain to be elucidated. A better understanding of astrocyte EAAT-2 regulation could lead to novel therapeutic options targeting astroglial dysfunction during neuroinflammatory diseases including HAND and METH abuse.

Another consequence of astrocyte exhaustion during chronic neuroinflammation is dysregulation of the tissue inhibitor of metalloproteinase (TIMP):MMP balance. Four TIMPs regulate MMP, enzymes that affect BBB integrity by altering the extracellular matrix. TIMP-1 is the only inducible member of the TIMP family of four inhibitors (Brew et al., 2000). Interestingly, CSF and brain tissue samples from HAD patients showed reduced TIMP-1 and increased MMP-2 levels compared to seronegative controls (Suryadevara et al., 2003). However, astrocytes upregulate TIMP-1 expression during acute IL-1β activation, HIV-1 gene expression or exposure (Suryadevara et al., 2003; Dhar et al., 2006; Fields et al., 2011). It is only during chronic activation that the astrocyte TIMP-1 expression falls, while expression of some MMPs is sustained, or augmented by infiltrating PBMC (Suryadevara et al., 2003; Chao and Ghorpade, 2009). TGF-β 1/2, an anti-inflammatory cytokine, decreases acute TIMP-1 expression in IL-1β-activated astrocytes. In contrast to TIMP-1, TGF-β 1/2 levels are increased in HAD brains compared to controls, thus TGF-β may contribute to TIMP-1 depletion during chronic neuroinflammation (Dhar et al., 2006). Since TIMP-1 also protects human neurons from HIV-1-induced apoptosis, decreased TIMP-1 expression also contributes to increased neurotoxicity due to diminished neurotrophic support (Ashutosh et al., 2012). TIMP-1 and other pro-inflammatory cytokine levels remained significantly elevated in rat striatum 24 h after HIV-1 Tat and METH injections, compared to either alone or vehicle (Theodore et al., 2006a). Repeated METH exposure increases MMP-2 and MMP-1 expression, which in turn enhances dopamine release and reward. The METH-mediated alterations in dopamine signaling and receptor expression were significantly attenuated in MMP-2 and MMP-9 KO mice, indicating that the MMP/TIMP system also regulates METH-induced behavioral sensitization (Mizoguchi et al., 2007a,b, 2008).

### Common signaling pathways

A large majority of bioactive molecules discussed above facilitate communication among various CNS cells.

Signals received by target receptors regulate astrocyte function during HIV-1 and METH-associated neuroinflammation through a variety of cross-linking pathways. As IL-1β is a prototypical cytokine for astrocyte activation, the NF-κB pathway contributes to the regulation of many astrocyte genes and is involved in cellular responses to stimuli such as stress, cytokines, free radicals, glutamate or viral antigens (reviewed in Mémet, 2006). Downstream of the IL-1 receptor (IL-1R), the IκB kinase complex phosphorylates and degrades the NF-κB sequestering protein, IκBα, leading to NF-κB release. Persistent NF-κB activation is implicated in the prolonged induction of selective pro-inflammatory genes in human glial cells (Griffin and Moynagh, 2006). The mitogen activated protein family of kinases (MAPK), including extracellular signal-regulated kinases (ERK), c-Jun N-terminal kinases (JNK) and p38, also regulate many HIV-1- and METH-induced astrocyte responses, which often culminate in NF-κB-mediated gene transcription (Table 2). IL-1β signaling can also be negatively regulated by expression of inhibitory type IL-1R, IL-1R antagonist, soluble and decoy receptors. Dysregulation of the IL-1β system in the brain has been implicated in AD, MS and epilepsy (Garlind et al., 1999; Ravizza et al., 2006; Dujmovic et al., 2009) Cytokine receptors for IFNs and a few ILs are coupled to the JAK/STAT pathway. Here, JAK phosphorylation of various tyrosine kinases facilitates STAT dimerization and gene transcription. METH- and Tat-induced astrogliosis and GFAP expression are also regulated through STAT3 (Robson et al., 2014; Fan et al., 2015) Ligation of G-coupled receptors such as CXCR4 can differentially initiate downstream elements including cAMP and [Ca^2+^]_i_ to mediate function. CXCL12 and gp120 induce ERK 1/2 activation in human neurons, while only CXCL12 did so in astrocytes (Griffin and Moynagh, 2006). Induction of differential signaling pathways also influences HIV-1 gene transcription in astrocytes, where TGF-β-linked transcription factors, Smad3 and 4, interact with C/EBP-β to offset Tat-mediated LTR activity (Coyle-Rink et al., 2002).

A consequence of extended activation of neuroinflammatory signaling cascades is Ca^2+^ dysregulation in both glia and neurons. Intracellular Ca^2+^, when released from the ER, acts as a secondary messenger and regulates the activity of many enzymes, ion channels and cytoskeletal components. In astrocytes, [Ca^2+^]_i_ signaling is induced by activity in adjacent neurons, glutamate, ATP, METH and HIV (Banerjee et al., 2008; Reddy et al., 2012). Dysregulation of [Ca^2+^]_i_ is implicated in astrocyte Aβ-associated neurotoxicity and ischemia, through Ca^2+^-mediated glutathione depletions and voltage-gated Ca^2+^ influx (Duffy and MacVicar, 1996; Abramov et al., 2003). These various routes of Ca^2+^ signaling converge on a common pathway involving Ca^2+^ overload-induced mitochondrial dysfunction, including oxidative stress, cytochrome c release and injury or apoptosis in neurons and astrocytes alike (Stanika et al., 2009; Eugenin and Berman, 2013).

## Therapeutics to target astroglia

The various roles of astroglia in CNS pathology are only beginning to be defined and reactive gliosis is now well recognized as a ubiquitous feature of CNS pathologies. Astrogliosis is not a simple on or off switch, but rather a finely tuned continuum of molecular, cellular and functional alterations. These changes in gene expression and function can exert both beneficial and detrimental effects in the brain milieu, dependent upon the duration and context of the specific molecular signaling cascades. Glial activation and dysfunction are emerging as important targets during neuroinflammation (Jha and Suk, 2014). Astroglia actively participate in neurodegeneration through the loss of normal functions and gain of abnormal activities. The ever-expanding understanding of the mechanisms regulating these changes has the potential to identify many molecules that may serve as therapeutic targets for neuroinflammatory disorders including METH abuse and HAND (Table 3).

**Table 3:** **Therapies targeting astroglial activation and function**.

**Agent**	**Mechanism**	**Outcome**	**References**
7-nitroindazole	Neuronal NOS inhibitor	Neuroprotective, blocked METH-mediated dopamine and DAT depletion	Itzhak and Ali, 1996; Schulz et al., 1997
Bryostatin 1	Macrolide lactone from bryozoans, anti-cancer, memory enhancing	Anti-inflammatory and neuroprotective, decreased HIV and CXCL10-mediated neurotoxicity and PBMC chemotaxis	Mehla et al., 2012
Buprenorphine	Pain and opioid replacement therapy	Anti-inflammatory, decreased MO chemotaxis, decreased METH-mediated ROS in glia	Fitting et al., 2014; Carvallo et al., 2015
Celastrol	Quinone methide-triterpene from *Tripterygium wilfordii*: anti-oxidant and anti-inflammatory activities	Anti-inflammatory, decreased CCL2, CXCL8, CXCL10, ICAM/VCAM-1 Anti-oxidant, increased HO-1 and NRF-2	Allison et al., 2001; Zhu et al., 2010; Youn et al., 2014
Clomipramine or Imipramine	Tricyclic anti-depressant, serotonin and norepinephrine reuptake inhibitor	Anti-inflammatory, reduced glial NO, IL-1β and TNF-α release	Hwang et al., 2008
Copaxone (Copolymer1, Glatiramer acetate)	Multiple sclerosis therapy, myelin immune decoy	Anti-inflammatory, decreased TNF-α, IL-1β, iNOS and increased BDNF	Gorantla et al., 2007, 2008
EPPTB	N-(3-ethoxyphenyl)-4-pyrrolidin-1-yl-3-trifluoromethylbenzamide, TAAR1 antagonist/reverse agonist	Anti-inflammatory and neuroprotective, decreased cAMP signaling and EAAT2 reduction in astrocytes, reduced lymphocyte activation	Miller, 2012; Cisneros and Ghorpade, 2014
Fingolimod	Multiple sclerosis therapy, lymphocyte sequestering	Anti-inflammatory, Decreased astrocyte activation, sphingosine-1-phosphate, IL-17, IL-1, NO	Colombo et al., 2014
Flavonoids	Naturally occurring in foods, inhibition of phospholipase A2	Anti-inflammatory, anti-oxidant	Nanda et al., 2007
IFN-γ	Replacement therapy, plasma IFN-γ levels depleted upon METH exposure	Neuroprotective, prevented METH-mediated reductions in DAT	Hozumi et al., 2008
Indomethacin	Anti-inflammatory (COX-2 inhibitor/NSAID)	Anti-inflammatory, prevented METH-induced glial activation	Gonçalves et al., 2010,^5^
N-acetyl cysteine amide (NACA)	Thiol anti-oxidant	Anti-inflammatory, protected from HIV-1 Tat/gp120/METH-mediated BBB pathology	Banerjee et al., 2010
NS-398	COX-2 inhibitor	Anti-inflammatory, decreased Tat-induced CCL2, IL-1β, IFN-γ, iNOS	Flora et al., 2006
Propentofylline (PPF)	Xanthine derivative, glial modulator	Anti-inflammatory and neuroprotective, reduced METH-associated astrocyte activation, and METH reward pathway, increased astrocyte glutamate uptake, impaired reinstatement of drug seeking behavior	Narita et al., 2006; Tawfik et al., 2006; Sweitzer and De Leo, 2011; Jacobs and De Leo, 2013; Reissner et al., 2014
Raltegravir	HIV-1 integrase inhibitor	Anti-inflammatory and neuroprotective, decreased neurotoxicity, inhibited astrocyte growth in glia/HN cocultures	Tatro et al., 2014
Sativex®	Synthetic Cannabinoids	Anti-inflammatory reduces astrogliosis and accumulation of chondroitin sulfate proteoglycans in MS	Feliú et al., 2015
SN79	Sigma-1R antagonist	Anti-inflammatory, reduced METH-mediated astrogliosis, microgliosis, neurotoxicity, hyperthermia	Seminerio et al., 2012; Kaushal et al., 2013, 2014; Robson et al., 2013b, 2014
Sodium Benzoate (NaB)	Food preservative and metabolite of benzoic acid found in food	Anti-inflammatory, decreased iNOS, TNF-α, IL-1β	Brahmachari et al., 2009
WIN55,212-2	Synthetic Cannabinoid	Anti-inflammatory, anti-oxidant and neuroprotective	Sheng et al., 2005; Rock et al., 2007; Hu et al., 2013; Aguirre-Rueda et al., 2015

### US food and drug administration (FDA) approved medications

Medications already in use for non-HIV/METH/astrocyte related therapies have shown changes in HIV-1- or METH-associated neuroinflammation, glial activation and neurotoxicity. Tricyclic antidepressants, such as clomipramine, are cited in the 2015 WHO model list of essential medicines needed in a basic health system to treat anxiety and depressive disorders by inhibiting serotonin and norepinephrine reuptake^6^. However, in a recent study on microglia and astrocyte cultures both clomipramine and imipramine reduced NO, iNOS, IL-1β and TNF-α expression by inhibiting IκB degradation, NF-κB p65 translocation to the nucleus and phosphorylation of p38 MAPK (Hwang et al., 2008). When used in microglia-neuroblastoma cocultures, both antidepressants significantly reduced glia-mediated-cell death (Hwang et al., 2008).

Fingolimod, an immune modulating drug used to treat MS, decreases astroglial activation and NO production in response to sphingosine-1-phosphate (S1P), IL-1β and IL-17 (Colombo et al., 2014). Fingolimod modulates autoimmune lymphocyte release from the lymph node by agonizing the S1P receptor, and also prevents monocyte: endothelial interactions (Bolick et al., 2005; Baumruker et al., 2007). However, in astrocytes fingolimod also decreased IL-induced, NF-κB-mediated signaling and reduced neurotoxicity following transfer of conditioned supernatants from activated astrocytes. Further, in an experimental autoimmune encephalomyelitis mouse model, fingolimod hampered astrocyte activation and NO production (Colombo et al., 2014). These results indicate that fingolimod can traverse the BBB and/or decrease monocyte infiltration into the CNS, supporting it as a candidate to decrease glial activation during HAND. However, these positive effects on glia would have to be balanced with impaired lymphocyte maturation in the lymph node. Copolymer-1, another MS drug that serves as a myelin decoy, showed anti-inflammatory benefits in an HIVE mouse model, with decreased pro-inflammatory cytokine and iNOS expression, coupled with increased BDNF levels. Both microgliosis and astrogliosis were reduced with treatment, which correlated with diminished neurodegeneration (Gorantla et al., 2007, 2008). These and other glial modulating, MS drugs may warrant future therapeutic consideration for HAND.

Over-the-counter COX-2 inhibitors and other non-steroidal anti-inflammatory drugs are widely used to treat pain and inflammation by blocking prostaglandin activation. Regulation of astrocyte gene expression during HIV-1- and METH-associated neuroinflammation involves common signaling intermediates including NF-κB and prostaglandins. In mouse studies indomethacin, a potent COX-2 inhibitor prescribed to treat inflammatory disorders such as rheumatoid arthritis, prevented or diminished METH-induced glial activation. GFAP and CD11b immunoreactivity and TNF-α/TNFR1 protein levels were reduced. Indomethacin inhibited METH-induced glial activation and hippocampal neuronal toxicity, preserving beta III tubulin, calbindin and tau expression (Gonçalves et al., 2010). NS-398, a COX-2 inhibitor in clinical trials for gastric cancer, decreased Tat-induced iNOS, CCL2, IL-1β and IFN-γ expression in brain glia. NS-398 was more effective than pyrrolidine dithiocarbamate, a potent anti-oxidant and NF-κB inhibitor (Huang, 2005; Flora et al., 2006). Current FDA-approved drugs capable of inhibiting these pathways in astroglia, may effectively reduce gain of function pro-inflammatory responses and reduce brain inflammation, if expeditiously approved for off-label uses to treat HIV-1 CNS infection and possibly METH abuse.

### Naturally occurring glia modulators

Food additives alter glial neuroinflammatory responses by regulating NF-κB activation. Sodium benzoate (NaB), a food preservative and a metabolite of benzoic acid, occurs naturally in cinnamon, cranberries, prunes, plums and apples. NaB is designated as “generally recognized as safe” by the FDA^7^, and is used pharmaceutically to treat urea cycle disorders and schizophrenia (Häberle et al., 2012; Lane et al., 2013). *In vitro*, microglial pro-inflammatory responses to LPS, HIV-1 Tat or Aβ, as measured by iNOS, TNF-α, IL-1β and surface markers, were significantly reduced by NaB treatment. IL-1β-activated mouse astroglia showed reduced GFAP and iNOS expression with NaB treatment (Brahmachari et al., 2009).

Celastrol is a triterpenoid quinone methide derived from perennial plants belonging to the Celastraceae family. Celastrol has exhibited anti-oxidant and anti-inflammatory effects in microglia and astrocytes (Jung et al., 2007; Nakamichi et al., 2010; Boridy et al., 2012; Youn et al., 2014). In astrocytes, celastrol inhibited HIV-1 Tat-induced expression of ICAM-1/VCAM-1 and pro-inflammatory chemokines CXCL8, CXCL10, and CCL2 in a JNK MAPK, AP-1, and NF-κB dependent manner. Further, celastrol downregulated these pro-inflammatory mediators by inducing HO-1 expression and Nrf-2 activation, both anti-oxidant responsive genes (Youn et al., 2014). Celastrol stands out as a prime therapeutic candidate for targeting glial activation as it inhibits glial-mediated inflammation while upregulating anti-oxidant machinery (Nakamichi et al., 2010; Youn et al., 2014).

Another class of plant metabolites, known as flavonoids, are found in tea, red wine, dark chocolate, *Ginkgo biloba* and berries (Haytowitz^8^). Research into their potential broad health benefits against oxidative stress, inflammation, cancer and cardiovascular disease is currently ongoing; yet, no health claims have been approved by the FDA or European Food Safety Authority for use as pharmaceutical drugs (Agostoni et al., 2010). However, flavonoids such as silibinin have been shown to possess anti-HIV-1 and HCV effects in T-cells by blocking viral replication, cell activation and proliferation (McClure et al., 2012). Orally administered anti-oxidants, such as flavonoids, have the capacity to inhibit microglial migration, ROS and IL-1β production, AA- and COX-2-mediated inflammation and toxicity (Nanda et al., 2007; Chuang et al., 2014; Singh and Pai, 2015). Assessment of ROS/RNS-mediated post-translational modifications of brain proteins in the CSF and brain tissues may reveal biomarkers associated with HIV-1-neurodegeneration (Uzasci et al., 2013). Biomolecules available in food by targeted dietary changes or supplementation that exert both generalized anti-oxidant and anti-inflammatory effects could penetrate the brain and reduce glial activation.

Therapeutic cannabis has been proposed in management of common comorbidities of HIV-1 infection (Woolridge et al., 2005; Whiting et al., 2015). Dronabinol (Marinol®) is an FDA approved synthetic Δ9-tetrahydrocannabinol (THC) that has been used to treat ART-associated nausea, appetite reductions and wasting syndrome (de Jong et al., 2005; Badowski and Pandit, 2014). Studies in Canada and England indicate that 27–38.5% of HIV-1-infected individuals used cannabis on a regular basis and self-reported benefits include relief of anxiety or depression, improved appetite, pleasure and pain relief (Woolridge et al., 2005; Harris et al., 2014). The physiological endocannabinoid (eCB) system consisting of cannabinoid receptors (CBR) and their endogenous ligands, eCB, are expressed by neurons, microglia and astrocytes (reviewed in Woolridge et al., 2005; Navarrete and Araque, 2008; Oliveira da Cruz et al., 2015). Hippocampal tripartite synapse signaling between astrocytes and neurons involves CB1R, a G-coupled protein receptor. CB1R stimulation by neuronal eCB leads to increased Ca^2+^ levels, glutamate release and activation of NMDA receptors of pyramidal neurons (Navarrete and Araque, 2008, 2010; Rasooli-Nejad et al., 2014). Studies of memory impairments induced by exogenous CB exposure were unchanged in neuronal CB1R KO and abolished in astrocyte CB1R KO mice. Further, inhibition of NMDA receptors also blocked CB-induced memory impairment, implicating astrocyte glutamatergic signaling as a key player in memory and learning (Han et al., 2012). Activation of astrocyte connexin-43 hemichannels by eCB releases ATP, which upregulates microglial pro-inflammatory responses during CNS injury (Vazquez et al., 2015).

In chronic neuroinflammatory disease models of HAND, AD, MS and stroke, eCB exert anti-inflammatory and neuroprotective effects in the CNS (Schiavon et al., 2014; Aguirre-Rueda et al., 2015; Feliú et al., 2015; Hind et al., 2015). A CBR synthetic agonist, WIN55,212-2 (WIN), protects neurons from gp120-mediated damage (Hu et al., 2013). In IL-1β-activated astrocytes, WIN decreases pro-inflammatory expression of TNF-α, CCL2, CCL5, and CXCL10 (Sheng et al., 2005). In microglia, WIN inhibits HIV-1 replication and decreases gp120-induced superoxide production (Rock et al., 2007; Hu et al., 2013). METH administration also increases eCB and CBR expression in the brain, suggesting that they may participate in METH-mediated neurotoxicity and behavioral changes. CBR antagonists reduce METH-seeking behavior following METH cessation and protect dopamine terminals from damage in mice (Anggadiredja et al., 2004; Loewinger et al., 2012). However, Δ9-THC administration with METH reinstatement reduced subsequent METH-seeking behaviors (Anggadiredja et al., 2004). Pretreatment with Δ9-THC blocks METH-induced neurotoxicity and astrogliosis by decreasing neuronal NOS and TNF-α levels, and by preserving tyrosine hydroxylase expression (Castelli et al., 2014; Nader et al., 2014). Together these studies suggest that synthetic cannabinoids may reduce glial activation during chronic HIV-1- and METH-associated neuroinflammation and protect neurons.

### Propentofylline

Propentofylline (PPF), a phosphodiesterase and adenosine reuptake inhibitor has been studied as a therapeutic treatment for various dementias and glioblastoma (Frampton et al., 2003; Jacobs et al., 2012). Interestingly, PPF treatment blocks METH-induced astrocyte activation and attenuates the METH reward pathway in mice. Further, intracranial injection of METH-treated conditioned media from astrocytes, but not from microglia, enhanced METH rewarding effects; suggesting astrocyte-specific regulation of METH reward pathways (Narita et al., 2006). PPF has also been shown to impair reinstatement of cocaine seeking behavior, which was dependent upon GLT-1/EAAT-2 expression and function (Reissner et al., 2014). PPF therapy increases EAAT-2 expression in astrocytes and dampens pro-inflammatory cytokine levels (Tawfik et al., 2006; Sweitzer and De Leo, 2011; Jacobs and De Leo, 2013). Since dysregulation of astrocyte EAAT-2 expression and function is implicated in both HAND and METH abuse, PPF could potentially target astrogliosis-mediated excitotoxicity and propagation of the neuroinflammatory environment by glia.

### Receptor antagonists

Astrocyte activation during METH abuse leads to persistent increase in GFAP immunoreactivity and reactive phenotypes even months after cessation of METH abuse. Therapeutic targeting of METH signaling receptors in astrocytes may reduce astroglial activation and impaired astrocyte function. In-depth studies on neuronal METH receptors have led to significant insight into the addictive and euphoric effects of METH abuse. In astrocytes; however, there is a paucity of these investigations with few recent reports that document METH receptors on astrocytes (Cisneros and Ghorpade, 2014; Robson et al., 2014; Zhang et al., 2015).

During METH exposure, trace amine associated receptor 1 (TAAR1) modulates dopamine levels in the synapse by regulating DAT activity in neurons. Activation of TAAR1 by METH stimulates protein kinase (PK)A and PKC to phosphorylate DAT. It has been proposed through studies in TAAR1 KO mice that phospho-DAT either acts in reverse, effluxing dopamine into the synapse, or is internalized, preventing dopamine reuptake from the synapse (Miller, 2011). TAAR1 is also expressed in primary human astrocytes, lymphocytes, B-cells and is upregulated during activation with METH and pro-inflammatory mediators (Panas et al., 2012; Babusyte et al., 2013; Cisneros and Ghorpade, 2014). In astrocytes, TAAR1 is upregulated during METH/HIV-1 cotreatment. Further, astrocyte TAAR1 activation by METH increases cAMP levels and downregulates EAAT-2 expression and function, which may lead to excitotoxicity and neuronal dysfunction or death due to impaired glutamate clearance from the synapse by astrocytes (Cisneros and Ghorpade, 2014). METH-induced alterations in EAAT-2 expression and function were blocked by TAAR1 knockdown, implicating TAAR1 as a therapeutic target for astrocyte-mediated neurotoxicity during METH and HIV-1 neurodegeneration (Miller, 2012; Cisneros and Ghorpade, 2014). In lymphocytes, METH-induced phosphorylation of PKA and PKC could be significantly reduced by EPPTB, a selective TAAR1 antagonist/reverse antagonist (Miller, 2012; Panas et al., 2012). However, TAAR1 KO mice show increased sensitivity to METH as measured by striatal dopamine release and augmentation of METH-induced behaviors (Wolinsky et al., 2007; Lindemann et al., 2008; Achat-Mendes et al., 2012). TAAR1 overexpression in the neurons of transgenic mice decreased sensitivity to amphetamine, even with increased extracellular dopamine levels in the accumbens nucleus and serotonin in the medial prefrontal cortex (Revel et al., 2012). Interestingly, attenuation of TAAR1 activation with a selective partial antagonist, RO5073012, restored METH-mediated changes in locomotor activity. Therefore, constitutive or tonic activation of TAAR1 by natural agonists may regulate physiological monoamine activity in neurons (Revel et al., 2012). TAAR1 agonists also suppress hyperactivity and improve cognition in glutamate receptor deficiency models (Revel et al., 2011, 2013) and TAAR1 modulates cortical glutamate NMDA receptor function in TAAR1 KO mice (Espinoza et al., 2015). Thus, a balance between agonism of neuronal TAAR1 and antagonism of astrocyte TAAR1 will need to be further investigated to balance the neuroprotective benefits of TAAR1 targeting drugs.

Sigma receptors have also garnered much attention in the neurodegenerative disease field as they have been implicated in pathology of neurodegenerative conditions including AD, PD, stroke and METH neurotoxicity (reviewed in Nguyen et al., 2014). Sigma receptor 1 (σ-1R) antagonists have been shown to attenuate METH-induced neurotoxicity, hyperthermia and behavior changes (Matsumoto et al., 2008; Kitanaka et al., 2009, 2012; Smith et al., 2010; Kaushal and Matsumoto, 2011; Kaushal et al., 2011; Robson et al., 2013a). Only recently have σ-R been studied in METH-mediated brain gliosis, where METH-induced GFAP expression was abrogated in σ-1R KO mice compared to controls (Robson et al., 2014). Further, METH-exposure leads to a positive feedback regulation in astrocyte σ-1R expression that could be inhibited with σ-1R antagonist BD1047 (Zhang et al., 2015). SN79, a σ-1R antagonist, has also been shown to block microglial and astrocyte activation, reducing expression of pro-inflammatory cytokine expression following METH treatment (Robson et al., 2013b, 2014), further implicating glial σ-1R as a therapeutic target for neurodegeneration. While σ-1R do not have intrinsic signaling machinery, they appear to modulate the activity of Ca^2+^ channels and signaling molecules by translocation and protein-protein interactions to regulate diverse cellular functions, including intracellular Ca^2+^ signaling, oxidative stress response, mitochondrial function, transcriptional regulation and cell survival. In such, drugs targeting sigma receptors in neurons and glia have vast implications in neurodegenerative disease and drug abuse (reviewed in Nguyen et al., 2014).

## Concluding remarks: HIV-1, METH, and astrocytes at neuroinflammatory crossroads

**Figure 1:**
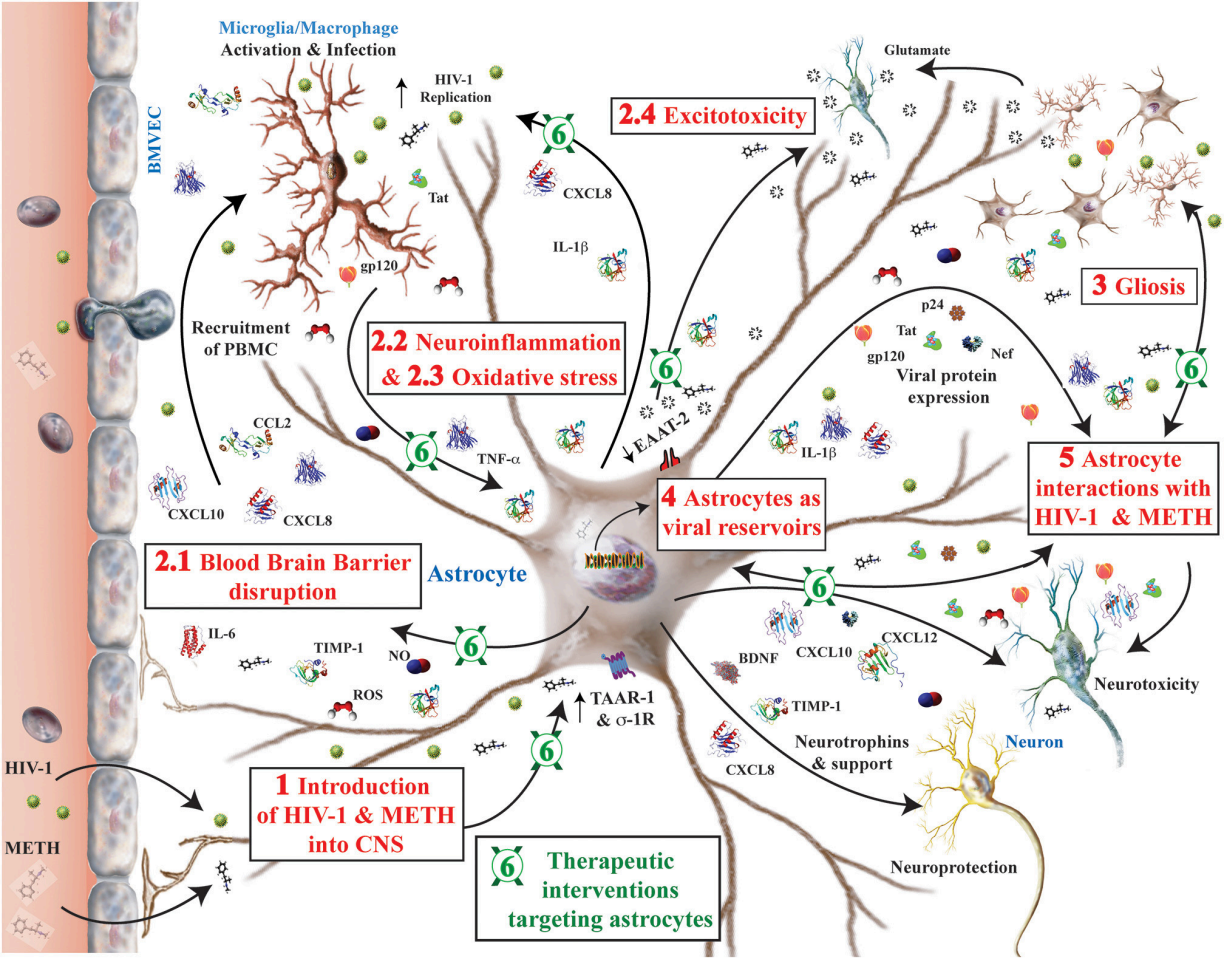
**An interactive neuroinflammatory roadmap crosslinking astrocytes with HIV-1 and METH**. The crystal structures of cytokines created using the data from Protein Data Bank (:PBD) for CCL2:1dok, CXCL8: 1IL8, CXCL10: 1o7z, CXCL12:1a15, IL-1β: 31BI, IL-6: 1ALU, TIMP-1: 1d2b TNF-α: 1TNF were rendered using PyMOL Molecular Graphics System (Schrödinger, LLC); BDNF: 1bnd, METH, ROS, NO were rendered using Accelrys Software (BIOVIA, San Diego, CA USA).

In this review, we have provided an in-depth summary of the existing literature about METH effects on astrocytes in the setting of HIV. This comprehensive overview indicates, without a doubt, that astrocyte regulation of neuroinflammation during HIV-1 infection and METH abuse involves a complex dialog between all neural cells. Figure 1 provides a graphic summary of ongoing events and a proposed temporal order for these activities. (1) As HIV-1 and METH gain access to the brain across the BBB, they interact with astrocytes and induce production of reactive oxygen and nitrogen species. (2.1) These along with cytokines and chemokines from either side of the BBB, act to increase BBB permeability. Chemokine gradients recruit leukocytes, which bring HIV-1 and inflammation as they extravagate into CNS. Brain microglia and perivascular macrophages, when activated and infected, secrete cytokines, virus, viral proteins and ROS, which in turn activate astrocytes to perpetuate (2.2) neuroinflammation and (2.3) oxidative stress. In response to activation, astrocyte EAAT-2 levels decrease and extra cellular glutamate levels rise. (2.4) Pathological glutamate levels overexcite neurons impairing function through excitotoxicity. (3) Concurrently, METH and neuroinflammation activate astrocytes and microglia in the vicinity, instigating reactive gliosis. (4) METH and other pro-inflammatory cytokines can activate proviral gene expression in astrocytes and microglia. (5) Infected glia secrete viral proteins and pro-inflammatory mediators, which alter astrocytes homeostatic functions and perpetuate neuroinflammation. Cytotoxic molecules, including cytokines, viral proteins and ROS, coupled with depletion of astrocytic neurotrophic support, induce neuronal dysfunction and death. (6) Intervening with therapeutics targeting astroglia may disrupt the neuroinflammatory dialogue and protect neurons during HAND and METH abuse.

Taken together, this comprehensive review further emphasizes that additional studies regarding glial-based mechanisms/interactions, implicated in the combined setting of METH and HIV, are timely and highly warranted. Moreover, this review presents a platform to persuade future investigators to examine several critical questions that remain unanswered and are likely to influence therapeutic outcomes. Perhaps, most importantly, it is yet unknown how these interactions differ in the setting of long-term ART. Are there any disparities related to the outcomes of the combined interplay outlined in Figure 1 in the setting of race and/or gender? Epigenetic factors may play a significant role in these phenomena and we have only begun to scratch the surface of the role of genetic background and/or predisposition. Over the next several years, HIV-associated comorbidities including neurological and metabolic complications and related astroglial contributions, will continue to hold high research priorities. While we have highlighted several salient features of astroglial contributions to neuroinflammation, the role of METH and other drugs of abuse in this setting will continue to unravel. Continued elucidation of the regulatory mechanisms governing astroglial responses to METH and HIV-1 will provide the foundation for the generation of novel therapeutic interventions for neuroinflammatory disorders by targeting a key player, astrocytes.

## Funding

The studies were supported by grant R01DA039789 from NIDA to AG.

### Conflict of interest statement

The authors declare that the research was conducted in the absence of any commercial or financial relationships that could be construed as a potential conflict of interest.

## Footnotes

^1^http://www.unaids.org/en/resources/campaigns/2014/2014gapreport/factsheet; http://www.cdc.gov/hiv/library/reports/surveillance/

^2^http://www.drugabuse.gov/publications/research-reports/methamphetamine/what-scope-methamphetamine-abuse-in-united-states

^3^http://stacks.cdc.gov/view/cdc/11778/

^4^http://www.drugabuse.gov/related-topics/trends-statistics/infographics/drug-alcohol-use-significant-risk-factor-hiv; http://www.drugabuse.gov/publications/drugfacts/hivaids-drug-abuse-intertwined-epidemics

^5^https://clinicaltrials.gov/ct2/show/NCT00172627

^6^http://www.who.int/selection_medicines/committees/expert/20/EML_2015_FINAL_amended_JUN2015.pdf?ua=1

^7^http://www.accessdata.fda.gov/scripts/cdrh/cfdocs/cfcfr/CFRSearch.cfm?CFRPart=184

^8^Haytowitz, D.B., Bhagwat, S., Harnly, J., Holden, J.M., Gebhardt, S.E. Sources of Flavonoids in the U.S. Diet Using USDA's Updated Database on the Flavonoid Content of Selected Foods. [Online]. Available: http://www.ars.usda.gov/SP2UserFiles/Place/80400525/Articles/AICR06_flav.pdf [Accessed 7/15 2015].
